# Home vision monitoring in patients with maculopathy: current and future options for digital technologies

**DOI:** 10.1038/s41433-023-02479-y

**Published:** 2023-03-27

**Authors:** Konstantinos Balaskas, Faye Drawnel, Arshad M. Khanani, Paul C. Knox, George Mavromaras, Yi-Zhong Wang

**Affiliations:** 1 Moorfields Eye Hospital NHS Foundation Trust, London, UK; 2 Institute of Ophthalmology, University College London, London, UK; 3 F. Hoffmann-La Roche Ltd, Basel, Switzerland; 4 The University of Nevada, Reno School of Medicine, Reno, NV USA; 5 Sierra Eye Associates, Reno, NV USA; 6 Department of Eye and Vision Science, University of Liverpool, Liverpool, UK; 7 Genentech Inc., San Francisco, CA USA; 8 Retina Foundation of the Southwest, Dallas, TX USA; 9 Department of Ophthalmology, UT Southwestern Medical Center, Dallas, TX USA

**Keywords:** Retinal diseases, Diagnosis

## Abstract

Patients with macular pathology, including that caused by age-related macular degeneration and diabetic macular oedema, must attend frequent in-clinic monitoring appointments to detect onset of disease activity requiring treatment and to monitor progression of existing disease. In-person clinical monitoring places a significant burden on patients, caregivers and healthcare systems and is limited in that it only provides clinicians with a snapshot of the patient’s disease status. The advent of remote monitoring technologies offers the potential for patients to test their own retinal health at home in collaboration with clinicians, reducing the need for in-clinic appointments. In this review we discuss visual function tests, both existing and novel, that have the potential for remote use and consider their suitability for discriminating the presence of disease and progression of disease. We then review the clinical evidence supporting the use of mobile applications for monitoring of visual function from clinical development through to validation studies and real-world implementation. This review identified seven app-based visual function tests: four that have already received some form of regulatory clearance and three under development. The evidence included in this review shows that remote monitoring offers great potential for patients with macular pathology to monitor their condition from home, reducing the need for burdensome clinic visits and expanding clinicians’ understanding of patients’ retinal health beyond traditional clinical monitoring. In order to instil confidence in the use of remote monitoring in both patients and clinicians further longitudinal real-world studies are now warranted.

## 摘要 摘要

为监测需要干预的疾病活动性和疾病进展, 年龄相关性黄斑变性和糖尿病性黄斑水肿引起的黄斑病变患者规律性临床随诊很有必要。到医院面诊的临床监测给患者、护理人员和医疗保健系统带来了重大负担, 其局限性在于它只能为临床医生提供患者疾病的瞬时情况。远程监测技术的出现为患者提供了在家中与临床医生合作监测自己视网膜健康的可能, 减少了门诊预约需求。在这篇综述中, 我们讨论了现有的和新的视觉功能测试, 这些测试具有远程使用的潜力, 并考虑了它们用于区分疾病存在和疾病进展的适用性。然后, 我们从临床开发到验证研究和真实世界的实施中回顾了支持使用移动应用程序监测视觉功能的临床证据。本综述确定了基于视觉功能测试的7种应用程序: 4种已经获得某种形式的监管许可, 3种正在开发中。本综述所包含的证据表明远程监测为黄斑病变患者在家监测其病情提供了巨大的潜力, 减少了繁琐的门诊需求, 并使临床医生对传统临床监测之外的患者的视网膜健康更为了解。为了使患者和临床医生对远程监测的使用有信心, 现在有必要进一步进行真实世界的纵向研究。

## Introduction

The effectiveness of vision-preserving treatments for patients with progressive diseases of the macula, such as age-related macular degeneration (AMD) and diabetic macular oedema (DMO), relies on timely detection of disease activity [1–3]. Early initiation of anti-vascular endothelial growth factor (VEGF) therapy can slow disease progression and preserve vision in patients with neovascular AMD (nAMD) or DMO [1, 3]. Regular monitoring of visual function and retinal health enables the timely detection of vision-threatening stages, such as the onset of nAMD, fellow eye conversion, or the detection of DMO in patients with diabetic retinopathy (DR) [1, 3–6]. However, retinal health must be assessed at outpatient retina clinics, hospitals, optometrist offices, pharmacies or specialist retina offices with ophthalmic imaging capabilities, principally optical coherence tomography (OCT) [1, 3]. The need for regular in-clinic assessments places a significant burden on patients, caregivers and healthcare systems and only provides snapshots of disease. Disease progression is unpredictable so, if patients are not monitored frequently enough, disease reactivation may only be detected after significant loss of vision has occurred [1, 7]. Conversely, non-treatment visits, which occur when disease activity is not detected during a clinic visit, are burdensome. Tailoring retreatment intervals to each individual case is crucial to ensure treatment frequency is sufficient to maintain disease control [7–10].

The coronavirus disease (COVID-19) pandemic has highlighted the potential of digital technologies to enable alternative models of care, with clinical applications of remote monitoring technologies emerging across a variety of medical disciplines [11, 12]. This has coincided with widespread smartphone ownership in the USA and Europe [13], with the majority of adults aged 55 years or over owning a smartphone [13, 14]. Indeed, globally, smartphones and tablets are already used extensively as aids by adults with visual impairments [15] and most patients with retinal disease report having both the means and motivation to engage in home monitoring [16].

Currently, remote monitoring for macular disease at scale can be achieved in a cost-efficient way only with respect to visual function monitoring. Miniaturisation of OCT imaging technology for home use has been investigated [17, 18]. However, given the extent of the at-risk population for degenerative macular pathology, technologies with a high upfront hardware cost, such as home retinal imaging devices, are unlikely to have sufficient reach in the near future. For patients with macular pathology, remote tests of visual function that can be administered via readily available devices, such as smartphones and tablets, have the greatest potential to benefit as many patients as possible. Although not a substitute for in-clinic, imaging-based monitoring, such remote monitoring has the potential to detect disease onset and disease reactivation. Consequently, remote monitoring could eventually be used to reduce non-treatment visits in *pro re nata* anti-VEGF regimens and to personalise treat-and-extend regimens. Reviews have identified over 40 apps that are available in the Apple and Android app stores for mobile or online visual acuity (VA) testing. However, they have also highlighted a lack of scientific validation and failure to meet the rigorous standards required for accurate testing of visual function in almost all these available apps [19–21]. To allay clinicians’ concerns regarding safety and effectiveness, remote monitoring solutions should be evidence based, developed in accordance with local medical device regulations and approved by regulatory authorities.

The purpose of this review was to identify available evidence on the performance and clinical utility of remote visual function monitoring mobile applications that may be used for at-home disease monitoring by patients with existing macular pathology.

## Methodology

This review includes literature meeting the following criteria: studies of remote visual function monitoring mobile applications, which may be installed on existing devices such smartphones or tablets, intended for self-monitoring use by patients with an existing macular pathology diagnosis. Technologies that were intended solely for screening of patients without existing pathology, required the use of bespoke hardware, did not require patients to self-test their vision (e.g., video consultation software), were not available on mobile devices or were only available via a web browser were not considered for this review.

We used Embase to identify relevant literature by searching titles and abstracts for the following keywords: visual acuity or sight test or vision test or hyperacuity; telehealth or telemedicine or remote or mobile application or smartphone or iPod; AMD or retinopathy(ies) or maculopathy(ies). Searches were limited to English language publications. There were no search limits on article types or publication date.

These searches were supplemented by our own knowledge of the relevant literature and with additional artificial intelligence-based techniques of semantic searching (www.semanticscholar.org) and citation searching of key papers (https://inciteful.xyz/). Table 1 provides a full description of the search strings used to identify the literature. These searches identified 104 papers. Of these, 21 papers reporting on seven apps met our inclusion criteria after abstract screening (Table 2) and are included in this review.

**Table 1:** Search terms used to identify the literature.

Search step	Search terms	Hits	New papers identified
Papers suggested by authors	N/A	5	N/A
Embase search	(“visual acuity” or “sight test” or “vision test” or hyperacuity).ti.ab AND (telehealth or telemedicine or remote or “mobile application” or “smart phone” or ipod).ti,ab AND (AMD orretinopath$ or maculopath$).ti,ab.	100	N/A
Semantic search (www.semanticscholar.org)	remote monitoring visual function “degenerative maculopathies” (filter: medicine)	64	2
Citation searching (https://inciteful.xyz/)	Citation searching of the five key papers identified by authors in step 1	N/A	2

*N/A* not applicable. All searches were limited to English language publications.

**Table 2:** Summary of apps identified in the literature search.

	OdySight	Verana Vision Test (previously Checkup)	Home Vision Monitor (previously myVisionTrack®)	Alleye	MultiBit Test	Central retinal sensitivity	Hyperacuity app
Approved as SaMD	FDA class 1 medical device and CE marked	FDA class 1 medical device^a^	FDA approved and CE marked^b^	FDA approved and CE marked	No	No	No
Rx only	Yes	Yes	Yes	No	N/A	N/A	N/A
Function tests employed	Visual acuity (tumbling E), contrast sensitivity (Landolt C), Amsler grid	Visual acuity (letter identification), Amsler grid	SDH	Alignment hyperacuity	Rarebit perimetry	Retinal sensitivity to luminance	Line distortion hyperacuity task
Parameters assessed	Visual acuity, contrast sensitivity, metamorphopsia/scotoma	Visual acuity, metamorphopsia/ scotoma	Hyperacuity	Hyperacuity	Visual field integrity	Visual field integrity	Hyperacuity
Target population	Patients with chronic eye diseases	Patients with AMD or DR	Patients already diagnosed with maculopathy, including AMD and DR	Patients already diagnosed with AMD	Patients with macular disease	Patients with AMD	Patients with AMD at risk of conversion to nAMD
Fixation point used during testing	Yes – Amsler grid fixation point	Yes – Amsler grid fixation point	No – test involves global visual integration	Yes – alignment of flanking dots judged relative to central fixation point	No – test involves global visual integration	Yes – targets varying in luminance presented around a central fixation point	No fixation point, but users must tap the centre of screen between trials
Citations	Brucker et al. 2019 [49] Nikam et al. 2020 [50]	Khurana et al. 2021 [51]	Wang et al. 2002; 2011; 2013; 2014; 2015 [24, 43, 61, 62, 64] Kaiser et al. 2013 [56] Ku et al. 2016 [55] Pitrelli Vazquez et al. 2018 [59] Korot et al. 2021 [57] Lott et al. 2021 [58] Schneck et al. 2021 [60]	Schmid et al. 2019 [23] Chong Teo et al. 2021 [66]	Winther et al. 2015 [48] Winther et al. 2015 [25]	Wu et al. 2015 [52] Ho et al. 2018 [53]	Chen et al. 2016 [22]

*AMD* age-related macular degeneration, *CE* Conformité Européenne*, DR* diabetic retinopathy, *FDA* Food and Drug Administration, *N/A* not applicable, *nAMD* neovascular age-related macular degeneration, *Rx* prescription, *SaMD* software as a medical device, *SDH* shape discrimination hyperacuity.

^a^Although the Verana Vision Test app itself has not been approved, all the tests within it have FDA clearance.

^b^The Home Vision Monitor app itself has not been approved, but the SDH test that it houses has FDA and CE clearance.

### Use and utility of mobile applications for remote monitoring of visual function

#### Visual function tests with potential for remote use

VA, visual distortion (metamorphopsia) and blind spots (scotomas) are routinely monitored as assessments of macular pathology. Contrast sensitivity assessments may also be monitored during in-clinic testing but are more frequently used in a research setting. Novel tests of two alternative aspects of visual function – hyperacuity and visual field damage – have also been developed to overcome some of the limitations of conventional measures when trying to sensitively measure subtle changes in visual function due to macular pathology [22–25].

##### Visual acuity

VA tools such as the Snellen chart and Early Treatment for Diabetic Retinopathy Study (ETDRS) chart measure an individual’s ability to recognise letters at high luminance and contrast at a set distance. However, many patients with diagnosed macular pathology have preserved VA during the early stages of the disease [26, 27]. By the time reduced VA is observed, the disease may already have reached an advanced stage. For example, VA assessed with a high-contrast Bailey–Lovie chart was insufficient to distinguish between patients with early DR, patients with diabetes who had not yet developed DR and people without diabetes [27, 28].

##### Metamorphopsia and scotoma

The Amsler grid is a rudimentary, long-established method for detecting signs of visual function deficits such as metamorphopsia and scotoma in patients with retinal diseases. The grid is a 10 × 10 cm chart with 400 single squares, each representing an angle of 1°. Patients are required to fixate on a central dot and report sections of distortions, blurring or missing lines on the grid [29]. Although the Amsler grid may have some utility in screening for retinal disease, it does not provide a precise, quantifiable assessment of visual function making it ill-suited for monitoring disease progression [29, 30]. The body of evidence supporting the use of the Amsler grid for screening or monitoring is relatively small [31]. A meta-analysis has revealed large variability in the sensitivity and specificity of the Amsler grid for screening in AMD [32]. Performance of the Amsler grid may also have been artificially boosted by the high proportion of case-control studies identified in this meta-analysis [32]. When monitoring established disease, the Amsler grid cannot reliably distinguish between metamorphopsia and scotoma, is unable to detect scotomas in almost half of eyes and is particularly insensitive to scotomas of 6° or less [33]. The Amsler grid’s appeal may lie in the lack of validated alternatives and the fact that it is easy to distribute and does not cost anything to administer, making it accessible to all patients [31]. However, despite its relative simplicity, performing an Amsler assessment correctly—including maintaining accurate and steady fixation, testing at an appropriate distance and correctly conveying the percept of the grid—can be deceptively challenging for patients [31]. Furthermore, real-world compliance with Amsler testing is poor, thus limiting its clinical utility [31, 34–36].

##### Contrast sensitivity

Contrast sensitivity is expected to decrease with the progression of retinal diseases, as neuronal dropout reduces the available pooled responses of retinal neurons to contrasting stimuli such as letter strokes of low-contrast letters [31]. Deficits in contrast sensitivity may distinguish patients with early-stage maculopathies, including AMD and DR, before VA is affected. However, contrast sensitivity does not distinguish between stages of disease progression and has little prognostic value, meaning that it is not well suited to disease monitoring [27, 37–39].

##### Hyperacuity

Hyperacuity is the ability to detect small spatial differences in alignment, orientation, position, curvature or circle modulations [40–42]. Of these, alignment and circle modulations have been the focus of research efforts to develop hyperacuity tests.

Alignment hyperacuity, also known as Vernier hyperacuity, requires patients to identify misalignment in stimuli along vertical, horizontal or oblique axes or to distinguish deviations in radial forms. Unlike in VA tasks, identification of such misalignments requires pooling of visual signals, especially for the tasks such as radial deformation detection, from across the fovea. A large portion of the retina must be healthy to process shapes optimally [43]. Thus, compared with VA performance, impairment in hyperacuity performance may indicate damage to a larger portion of the central retina [23, 40, 42]. The Shape Discrimination Hyperacuity (SDH) task detects early functional deficit in macular pathology before VA is affected, with minimal interference from normal ageing [43]. Humans with healthy vision have a high sensitivity to detect sinusoidal distortions in circular stimuli [42]. The SDH test requires patients to distinguish a circle that is distorted by radial modulations from perfect circles. The test captures the minimal amplitude of radial modulation that allows the individual to correctly identify the distorted circle [43]. Unlike VA tests, hyperacuity tests such as the SDH test are not limited by photoreceptor spacing and are less affected by optical filtering, which means that the test may be more accurate than VA tests in revealing dysfunction of the neural retina and can detect visual disturbances that result from distortion of the photoreceptor layer caused by fluid accumulation [43–45].

##### Visual field integrity

Microdot perimetry methods test photoreceptor damage across multiple points in the visual field. Such methods can detect localized points of damage in the retina that may not be detected with foveal visual function assessments. Rarebit microdot perimetry uses brief exposure to high-contrast microstimuli to test visual field damage at numerous separate locations on the retina and quantify photoreceptor degradation. The proportion of missed targets reflects the extent of neural depletion [46]. Rarebit microdot perimetry has shown promise in identifying patients with early-stage diabetic maculopathy and AMD [46–48].

#### Implementing visual function tests for remote monitoring

The literature search identified seven app-based visual function tests, of which four have received regulatory clearances in the USA and/or Europe, with three in development or being assessed in clinical studies (Table 3). Of these apps, two implement digital versions of conventional in-clinic tests and five implement novel tests of hyperacuity or visual field integrity.

**Table 3:** Summary of published results available for each app.

	OdySight	Verana Vision Test (previously Checkup)	Home Vision Monitor (previously myVisionTrack®)	Alleye	Multibit test	Central retinal sensitivity	Hyperacuity app
Reliability							
Test–retest analyses	NA	NA	Test results stable in healthy eyes within each session and over time and in eyes with AMD/DR without clinical progression over a 6-month period [55, 62]	NA	NA	Acceptable repeatability for AMD and fellow eyes [53]	NA
Convergent validity							
Convergence with in-clinic or standard at-home tests (e.g., VA, Amsler grid)	VA and Amsler app assessment converges with clinic tests, contrast sensitivity underestimated visual function [49]	VA and Amsler app assessment converges with clinic tests in eyes with AMD/DR [51]	SDH correlates with VA in eyes with AMD or DR [24]	NA	NA	No significant difference between retinal sensitivity assessed with the app or with microdot perimetry [52]	Test correlates with Snellen assessment of VA [22]
Convergence with imaging measure ofdisease pathology	NA	NA	SDH worsens asCST increases andSDH significantlyworse in eyes withlarge drusen andRP abnormalities vsonly large drusen inpatients with AMD [24, 60, 64]	NA	The test hadgreaterconvergence withphysician assessment of disease progression than VA measure [48]	Retinal sensitivity changes in line withunderlying pathological changes (presence of atrophy, presence of drusen,RPE disruption and absent ellipsoid zone) [53]	Significantly higher scores in eyes withhigher RPE irregularity [22]
Diagnostic performance							
Discrimination of presence of macular pathology	NA	NA	SDH significantly worse in eyes with AMD than in healthy eyes [43] SDH significantly worse in participants with intermediate AMD vs healthy controls [58]	App discriminates between eyes with nAMD and healthy controls [23]	Test discriminates between AMD and healthy controls [25]	NA	NA
Discrimination of disease stages	NA	NA	SDH significantly differs among stages of AMD and DR [24, 64] SDH distinguishes between intermediate vs early AMD [58]	App discriminates between eyes with nAMD and dry AMD [23]	Test discriminates between nAMD and dry AMD [25]	NA	High sensitivity and specificity for distinguishing between nAMD requiring treatment and stable nAMD or dry AMD [22]
Detecting onset of new disease	NA	NA	Numerically higher sensitivity and specificity than ETDRS and Amsler grid at differentiating conversion to nAMD from moderate AMD [64] Moderate sensitivityfor detecting nAMD in fellow eyes [59]	NA	NA	NA	NA
Detecting clinical changes during anti- VEGF therapy	NA	NA	Clinical improvements after 3 and 6 months of therapy accompanied by improvements in SDH in patients with DR [61] 84.6% sensitivity and 88.5% specificity for detecting vision change in patients receiving anti- VEGF treatment	NA	NA	NA	NA
Clinical utility							
User satisfaction	NA	94% of patientswith AMD or DRwould continue to use the app if it were available [51]	98.4% of patients with nAMD reportedthe SDH test waseasy or very easy to use [57]	NA	NA	NA	90.9% of patients rated test as easy to use [22]
Compliance	Retention rate of 43% at 3-month follow-up [50]	100% of patients with AMD or DRwere compliant with twice-weekly testing over 3-month period [51]	64% compliance with at least twice- weekly testing for at least 4 continuous weeks among patients with nAMD [57]	43% compliance with twice-weekly use among patients with retinal pathology [66]	NA	NA	NA

*AMD* age-related macular degeneration, *CST* central subfield thickness, *DR* diabetic retinopathy, *ETDRS* Early Treatment in Diabetic Retinopathy Study, *NA* not available, *nAMD* neovascular age-related macular degeneration, *RP* retinal pigment, *RPE* retinal pigment epithelium, *SDH* shape discriminationhyperacuity, *VA* visual acuity, *VEGF* vascular endothelial growth factor.

##### Apps that implement digital versions of conventional in-clinic tests

OdySight is a prescription-only app utilising gamified visual function tests of near VA, contrast sensitivity and metamorphopsia and scotomas. Modules include a test of near VA based on the ETDRS, in which patients must identify the direction of a tumbling letter E, a contrast sensitivity task based on the Pelli–Robson chart, in which patients indicate the direction of the Landolt C, and a digital Amsler grid, adapted so that the typical paper grid is divided into three parts to display across a smartphone screen. Patient scores on each test are sent to a secure server where they are accessible to the prescribing physician [49]. The app has been tested in 1028 participants in two studies assessing its validity and real-world utility.

In an open-label, single-arm, prospective study, the OdySight app was validated in a controlled laboratory setting against the traditional physical tests that the app modules emulate. Participants (*n* = 78, 120 tested eyes) were recruited from the French healthcare system and included those with and without established eye disease (the most common diagnoses being AMD, retinitis pigmentosa, Stargardt disease and glaucoma). All tests were performed on iPhones at a distance of 40 cm, using an ophthalmological chinrest, and with luminosity standardised using a lux metre.

OdySight uses the phone’s front-facing camera to ensure that participants complete the test at a standardised distance and luminosity when the app is used at home. This feature was not tested in the validation study. Bland–Altman analysis demonstrated no disagreement between the Sloan- near ETDRS (mean difference 0.53 letters [95% confidence interval (CI) −0.42, 1.48]) and the equivalent digital test on the app with a 95% limit of agreement (LoA) of −9.75 to 10.82 letters. When compared with the 4 m ETDRS there was an underestimation bias (mean difference −1.53 letters [95% CI −2.78, −0.27]; 95% LoA −15.16, 12.11). The digital contrast sensitivity (LogCS) module underestimated visual function by the equivalent of more than one line on the Pelli–Robson chart (mean difference −0.16 logCS [95% CI −0.20, −0.13]; 95% LoA 0.54, 0.22). There was no difference in the detection of metamorphopsia or scotoma between the app-based and the paper Amsler grid (McNemar test *p* = 1.0 for both comparisons). Participants undertook a training session before enrolment; three individuals were excluded from participating because they were unable to correctly use the app. Nonetheless, investigators noted some difficulties that participants had when using the app, including trembling hands when using touchscreens and difficulty understanding instructions [49]. Data on the real-world use of the app since September 2018 have also been reported in 950 patients who were recommended OdySight by their ophthalmologists for chronic eye conditions, including AMD, DR, venous thrombosis and other conditions [50]. The app has been most commonly recommended for patients with nAMD, which accounts for 35% of patient downloads. These patients were also the most active users of the app. No data were reported on whether OdySight was recommended by ophthalmologists for the detection of disease or the monitoring of treatment regimens. Most users (74%) of the app were aged 50–80 years, with patients aged 70–80 years having the highest engagement rate (exact engagement rate was not reported). However, even the oldest patients (>80 years old) had an engagement rate of over 50%. Three months after initiation, 43% of the overall sample and 43% of patients aged 80 or older continued to use the app.

Verana Vision Test (previously Checkup) is a prescription-only mobile application incorporating VA and Amsler assessments intended for home monitoring of patients with AMD and DR. To test near VA, the app presents a series of letters one at a time in progressively decreasing font sizes. The patient must identify the correct letter from a range of options presented in large font size beneath the target letter. It also includes an Amsler grid assessment of metamorphopsia [51]. In a prospective, single-arm observational study with 108 participants, the app was validated against reference tests (the Lebensohn Near Card for VA and the paper Amsler grid for metamorphopsia) to assess test–retest reliability in the clinic and at home. Participants with AMD, DR or normal vision (healthy controls) attended the clinic at baseline and at 1- and 2-month follow up [51]. During every clinic visit, participants completed two assessments each of the app and reference tests. Between clinic visits, participants were instructed to use the app at least twice weekly at home. Although the mean VA of the entire cohort was not reported, correlation plots indicated the majority of participants had VA of 0.5 logarithm of the minimum angle of resolution (LogMAR) or better, which is not representative of typical patient cohorts with late AMD (neovascular or non-neovascular). Canonical correlation (rc) plots indicated good correlation between the app-based and reference VA assessment in the dominant cluster of cases with VA better than 0.5 LogMAR, while poor correlation was inferred from the limited number of cases with worse vision (rc = 0.857). App-assessed VA appeared to be underestimated when compared with the reference test (slope = 0.71). Critically, when the Verana Vision Test was compared with the reference VA, the lower LoA of 0.3 LogMAR in Bland–Altman analysis was not met. Home versus clinic and test–retest values indicated good reproducibility (rc = 0.962 and rc = 0.901, respectively). The test–retest results were stated to have met the upper and lower LoA in Bland–Altman analyses (results not shown in the study publication). Bland–Altman plots, which are not reported, are described by the authors as the most reliable metric of agreement and were the basis of the primary analysis for this study. A limit of 0.3 LogMAR (equivalent to three lines on the ETDRS) is a substantial difference in visual function, which the authors considered clinically acceptable. The app-based and paper Amsler grid results for the presence or absence of metamorphopsia (assessed as a binary yes/no variable) were reported to be in strong agreement (sensitivity 93%, specificity 92%; CIs not reported). However, this binary assessment of Amsler testing does not provide direct evidence of how well this test will monitor disease progression. Participants reported being highly satisfied with the app’s usability and 94% expressed an interest in continuing to use the app if it were available. Compliance data supported participants’ subjective reports of satisfaction, as all patients used the app to test their vision at least twice a week for the 3-month study duration [51].

A project that is in the early stages of development, but that has the potential to be implemented as a tablet application, uses a visual field test to measure central retinal sensitivity to luminance in patients with AMD [52]. The task comprises a uniform background and target stimuli of white dots that vary in luminance level by increments of 1 dB. Target stimuli are presented randomly at five locations within the central 1° radius surrounding a central fixation point. Users indicate when they see the targets and thresholds are obtained using a staircase procedure. The task has been examined in two studies with a total of 83 participants [52, 53].

In validity testing of this task, presented in the clinic on an iPad, mean central retinal sensitivity was comparable when assessed using the app versus microdot perimetry (25.7 dB, standard error of the mean [SEM] 0.4 versus 26.1 dB, SEM 0.4; *p* = 0.094) in a sample of 30 patients with at least intermediate bilateral AMD [52]. Further investigation of this task in a sample of patients with nAMD being treated with anti-VEGF and a subset of at-risk fellow eyes found it to be a feasible task for use in an elderly patient population (*n* = 53, 74 eyes). Test–retest coefficients for the retinal sensitivity task were acceptable for both treated eyes (12.3 dB, 53 eyes) and fellow eyes (10.2 dB, 21 eyes). In treated nAMD eyes, the task was able to detect changes in retinal sensitivity as they related to underlying pathological changes of presence of atrophy, retinal pigment epithelium disruption and absent ellipsoid zone. In at-risk fellow eyes, retinal sensitivity changes were also associated with the presence of drusen. This task would, therefore, be suitable for monitoring disease progression in patients being treated with anti-VEGF and for detecting new disease onset in fellow eyes [53].

##### Apps that implement novel tests

The Home Vision Monitor (HVM) app utilises the SDH test, a prescription-only software-based medical device intended as an aid for monitoring disease progression in patients with macular pathology. HVM has been granted Food and Drug Administration (FDA) clearance in the USA, under its previous name myVisionTrack®, and is Conformité Européenne (CE)-marked in Europe. The SDH test is intended for the detection and characterisation of the central 3° metamorphopsia in patients with macular pathology, including those with AMD or DR, and as an aid in monitoring progression of disease factors causing metamorphopsia [54]. The current SDH test presents the user with four circles as stimuli, one of which is radially distorted, and asks the user to identify the distorted stimuli by touch (a four-alternative, forced-choice task) [55]. As the test progresses, the extent of radial distortion is reduced in a stepwise manner and a hyperacuity threshold measured in LogMAR is calculated based on the lowest detected level of distortion [56]. From its initial development as desktop software, the SDH has been adapted for patient-directed use on a handheld device (tablet or smartphone) and refined over time from a two-shape to four-shape alternative forced-choice test [24, 43, 55]. In the literature identified in this review, the SDH test has been tested in 1346 participants in 11 cross-sectional, prospective or real-world studies [43, 55–64]. These studies have addressed the reliability, convergent validity and performance of the test in distinguishing disease states and stages, and the real-world utility of the app.

In validity testing of the two-shape, desktop SDH test, SDH scores were significantly impaired in patients with early AMD (*n* = 20) compared with healthy controls (*n* = 10), even in the absence of VA deficit [43]. In a study of healthy volunteers (total *n* = 186), there was no significant variability between multiple tests taken within a single session (*n* = 74; mean [standard deviation (SD)] test difference = 0.02 [0.12] LogMAR) or between tests taken weeks (*n* = 30; mean [SD] test difference = 0.04 [0.2] LogMAR) or years (*n* = 15; mean [SD] test difference = 0.04 [0.18] LogMAR) apart, indicating good test–retest consistency over time. This study also examined alternative versions of the SDH test using a three-alternative and four-alternative forced choice (*n* = 106): both versions of the task had similar thresholds for detection of radial distortions [55]. The test–retest reliability of the SDH test has been confirmed in a sample of patients with AMD or DR (*n* = 44), with an average test variability of SDH measurements over 6 months of 0.098 LogMAR in eyes without clinical disease progression [62].

Concerning performance, when examined in a sample of nine eyes with nAMD and 24 eyes with high-risk moderate AMD, the SDH test had 88.9% sensitivity (95% CI 56.5, 98.0) and 79.2% specificity (95% CI 59.5, 90.8) for differentiating between disease states [64]. In a sample of patients with AMD (*n* = 37) and DR (*n* = 36), and healthy controls (*n* = 27), SDH test performance significantly discriminated between healthy controls and early, intermediate and advanced AMD (one-way analysis of variance [ANOVA], *p* < 0.0001) and between healthy controls and mild-to-moderate non-proliferative DR (NPDR), severe-to-very severe NPDR or pre-proliferative DR, and proliferative DR or NPDR affecting the fovea (one-way ANOVA, *p* < 0.0001). This study also found that SDH values significantly correlated with VA in eyes with AMD (*r* = 0.69, *p* < 0.0001, slope of 0.95) and eyes with DR (*r* = 0.66, *p* < 0.0001, slope of 0.75), and that declining SDH performance was associated with increasing central subfield thickness (*r* = 0.58, *p* < 0.0001) [24]. In a prospective, observational, longitudinal study of 179 patients with unilateral nAMD being treated with anti-VEGF, healthy fellow eyes were assessed using routine clinical assessment (OCT, VA assessment, dilated slit- lamp assessment) and the SDH test. In an analysis of the diagnostic performance of SDH in detecting new onset of nAMD in fellow eyes, the receiver operating characteristic (ROC) curve demonstrated that at a cut-off of –0.60 LogMAR, sensitivity was 0.79 (95% CI 0.54, 0.94) and specificity was 0.54 (95% CI 0.46, 0.62) [59]. A cross-sectional investigation of the predictive value of SDH in a sample of patients with early or intermediate AMD and healthy controls (*n* = 125) found SDH performance to be significantly associated with intermediate AMD, with a relative risk ratio of 2.34 (95% CI 1.24, 4.44) versus healthy controls and 1.82 (95% CI 1.04, 3.21) versus early AMD [58]. In a sample of 39 patients with bilateral intermediate AMD, mean SDH threshold was higher in eyes with large drusen and retinal pigment abnormalities than eyes with large drusen only (−0.40 ± 0.04 versus −0.61 ± 0.02; *p* = 0.001) despite there being no differences in VA between these groups [60]. Such results support SDH use in monitoring disease status to detect onset of new disease.

As part of an ongoing longitudinal study, 33 patients with DMO used the app alongside their usual anti-VEGF treatment. Clinical improvements, as judged by the treating retinal specialist, were accompanied by improvements in SDH at 3 and 6 months after initiating anti-VEGF treatment. These results suggest that the SDH test can detect improvements in disease status as a result of treatment and is, therefore, suitable for monitoring treatment outcomes [61]. In a prospective, single-arm, open- label pilot study, 160 patients with nAMD used HVM daily at home and during routine clinic visits for 16 weeks to assess the app’s usability and acceptability. Patients were encouraged to use the SDH test daily at home with no upper limit on testing frequency. Mean compliance (the proportion of days in which an HVM assessment was performed) was 84.7%. An average of 77.8% of patients used HVM on at least 80% of days and over 95% of patients tested at least weekly. In a patient survey, 92.5% of patients reported that the app was easy to use, with only one patient reporting difficulties in understanding how to operate the app [56].

In the first year of the COVID-19 pandemic, the HVM app was deployed at scale at a major tertiary care ophthalmology centre. Patients attending the clinic for anti-VEGF treatment for nAMD, macular retinal oedema caused by DMO or retinal vein occlusion, or another condition (*n* = 417) were prescribed the HVM app and instructed to self-test their vision twice weekly [57]. Uptake, defined as the patient installing the app and using it at least once, was favourable, with an uptake rate of 62% (258/417 patients). Compliance was also high: 64% of active users (166/258 patients) met the criteria for compliant use (any continuous period of at least 4 weeks in which the patient used the app at least twice weekly). Mean (SD) use rate of the app was 1.83 (2.46) uses per week at week 4. Almost all patients (92.3%) gave a satisfaction rating of 3 or more out of 5. Similarly, patients reported that app use met their expectations (80.3% rating 3 or higher). Most patients would recommend the app to others (88.5%) and found the app easy or very easy to use (98.3%). The majority of patients (69.9%) reported a high level of comfort in using digital technologies, and this comfort level was strongly associated with greater patient engagement [57].

An ongoing longitudinal, real-world study (MONARCH)is investigating the ability of a number of tests, including HVM, to detect reactivation of nAMD. Patients will use HVM for at least 12 months, alongside their routine care, and self-monitoring assessments will be compared against the reference standard of diagnosis of active disease [65].

The Hyperacuity App is an iPad app intended to quantify visual defects that indicate progression of nAMD [22]. The app uses a hyperacuity task in which patients must identify modulations on a straight line. A vertical or horizontal line is presented on screen and the user must identify the location of an arced distortion presented on the line for 200 ms. Some trials are randomised to have no distortion. Between trials the patient must select the centre of the blank screen to refocus the fovea on the centre. A user score is generated based on a grading system optimised to detect errors in distortion perception. The authors did not provide any further details about this grading system. In a cross-sectional study of 33 patients (53 eyes) with AMD of any stage, the app had sensitivity of 92.3% (95% CI 62.1, 99.6) in distinguishing between patients with dry AMD and those who did and did not require treatment for nAMD, as determined by an OCT exam (sensitivities were the same across all group comparisons). The app had specificity of 61.5% (95% CI 44.7, 76.2) in distinguishing between patients who required treatment for nAMD and those who did not (stable nAMD or dry AMD). Specificity was 43.8% (95% CI 20.8, 69.4) for detecting nAMD that did and did not require treatment and 73.9% (95% CI 51.3, 88.9) for differentiating between nAMD requiring treatment and dry AMD. Scores on the app were positively correlated with VA assessed with a Snellen chart, with unranked Pearson/Spearman correlations being 31.52%, 32.04% and 57.10% for dry AMD, nAMD not requiring treatment and nAMD requiring treatment, respectively. App scores were also significantly higher in eyes with higher retinal pigment epithelium irregularity as graded by OCT exam (one-way ANOVA, *p* < 0.0001). Three patients were excluded before testing as they were unable to use the iPad. On completion of testing, 90.9% of patients rated the test as being easy to use [22].

Alleye is a CE-marked and FDA-cleared mobile app indicated for monitoring of disease progression in patients with AMD via the detection and characterisation of metamorphopsia using a monocular alignment hyperacuity task. The app is available without prescription for patients to use on their own smartphones. Users are required to align a mobile central dot with two fixed flanking dots to form a straight line. The task is repeated three times with differing distances between the fixation and flanking dots in the horizontal, vertical and oblique axes. The task applies a similar principle to detect metamorphopsia as the Amsler grid but ensures that the fovea remains fixed on the central fixation point. While the user fixates on the central point, the test examines the areas of the macula where the flanking dots are presented. Unlike the Amsler grid, this task is able to give a quantitative measure of visual function [23].

Alleye has been tested in two studies examining diagnostic performance and real-world utility (*n* = 874). In a cross-sectional, in-clinic investigation of the diagnostic accuracy of Alleye, patients with varying degrees of AMD (89 eyes) and healthy controls (53 eyes) self-administered Alleye tests following a routine ophthalmological examination. The app had good diagnostic performance in discriminating between patients with nAMD and age-matched healthy controls (area under the ROC curve [AUC] = 0.845, 95% CI 0.759, 0.932) and in discriminating between nAMD and AMD without choroidal neovascularisation (AUC = 0.660, 95% CI 0.520, 0.799), making it suitable for detection of new disease onset [23].

The real-world utilisation of Alleye has also been examined in the context of disruption to in-person clinic visits caused by the COVID-19 pandemic. In an observational study, patients at a specialist retinal clinic were offered use of self- monitoring in place of deferred clinic appointments during the Singapore lockdown period. Owing to the extenuating circumstances of lockdown, patients were not given any in-person training with the app and were instead invited to participate via SMS text messaging. Patients had a variety of diagnoses, including AMD, DR, DMO, retinal vein occlusion and other retinal pathology. Uptake and compliance with self-monitoring were both low. Of 2774 patients who were contacted, only 26% (732 patients) signed up for self-monitoring. Of those signing up, 43% (315 patients) used the app at least twice weekly and were defined as compliant. Younger patients and those living with family or friends were significantly more likely to sign up for home monitoring (both *p* values < 0.05). Compliance was highest in patients with AMD, particularly those who had already lost substantial vision in their worse-seeing eye, suggesting greater motivation for self-monitoring in patients with experience of vision loss that requires active treatment and monitoring.

The low uptake and compliance in this sample emphasise the importance of comprehensive support in introducing patients to remote monitoring and training them in the use of apps. Among patients who were compliant in their app use, there were 33 trigger events indicating potential disease progression (defined as three test scores at least 25 points below the patient’s reference score). Of these triggers, 79% were attributed to incorrect app use, including testing of the wrong eye, not testing at the prescribed interval and patient-reported difficulties in using the app. Of the remaining seven triggers, patients were given urgent clinic appointments and 5/33 (15%) of the total triggers were confirmed as disease progression. In the sample of 315 compliant users, this translated to a false positive rate of 9% and a true detection rate of 1.6%. The authors asserted that they considered this false positive rate to be acceptable in the context of allowing deferral of appointments in the 89.5% of patients who did not have a trigger event during the study [66].

The MultiBit Test (MBT, previously DigitStep) utilises rarebit perimetry to examine the degree of disease severity in patients with AMD. In this task, multiple rarebit stimuli are presented in a pattern to form segmented numerical digits. The stimuli are presented with a varying number of rarebits per segment (RPS), until the minimum number of RPS required for pattern identification is found. Users with greater degrees of receptor loss in the neuroretinal field require a greater number of RPS to identify the stimuli [25]. Two studies with 110 participants have examined the convergent validity and diagnostic performance of the MBT. In initial in-clinic testing of the desktop version of the task in patients with drusen, dry AMD and nAMD, and healthy controls (*n* = 62), the MBT had an estimated sensitivity of 91% (AUC = 0.95, 95% CI 76, 94) at 90% specificity for detecting presence of disease. In distinguishing between dry AMD and nAMD, the MBT had an estimated sensitivity of 64% (AUC = 0.71, 95% CI 34, 83) [25]. The MBT was subsequently adapted as an app for use on iPhones and iPads and trialled in a longitudinal pilot study in patients with varying degrees of nAMD and healthy controls (*n* = 48) over an average of 39 weeks. MBT assessments showed good concordance with disease progression as assessed by clinical examination (kappa = 0.41 [95% CI 0.29, 0.53]). MBT scores remained stable during times of clinical stability, improved following successful anti-neovascular treatment and deteriorated during times of clinical recurrence [48]. The MBT is also currently under large-scale longitudinal investigation as part of the MONARCH trial to estimate the accuracy of the MBT and other apps as home monitoring tools for detecting the reactivation of nAMD [65].

## Discussion

This review aimed to identify and examine extensively the latest evidence on the reliability, validity, performance and clinical utility of remote monitoring mobile technologies that could be used to monitor disease status and progression in patients with existing macular pathology. Numerous promising apps for remote monitoring of visual function are in development or already available to patients and clinicians. Remote monitoring vision tests offer the potential to detect early signs of disease progression occurring between clinic visits and help identify the patients who need to be seen in-person. However, apps must be capable of detecting subtle changes in visual function, and they must be simple and easy to use. These are prerequisites for acceptance and use by healthcare providers and by patients, many of whom will be elderly and have impaired vision.

For patients with macular pathology, there is an unmet need to improve monitoring paradigms to reduce burden on patients and identify disease progression in a more efficient manner. While digitising conventional in-clinic tests, such as VA charts and the Amsler grid, will make them more readily available, doing so does not address their inherent limitations when performed in the clinic [27, 28, 31]. However, evidence indicates that quantitative tests that assess hyperacuity or visual field damage, which are not dependent on the limits of resolution acuity, could be more sensitive than VA and Amsler grid measures to differentiate the stages of disease, including pathologies that warrant treatment, such as nAMD [23, 24, 46–48]. Although it should be noted that visual field testing may be sensitive to refractive error and the presence of cataracts [67, 68]. The small, but growing, body of literature examining the use of remote monitoring for patients with maculopathies indicated good acceptability to patients and confirmed the general feasibility and validity of the remote monitoring approach. Already, at least two clinical teams have been able to implement remote monitoring during the COVID-19 pandemic [57, 66]. A central benefit of remote monitoring technologies is that they can be widely accessible for large numbers of patients. High rates of smartphone ownership mean that remote monitoring can be rolled out to the substantial population who are at risk for macular pathology with little or no upfront cost for the patient. Although hardware-based home monitoring systems for retinal health do exist—for example, ForeseeHome (Notal Vision Inc., Manassas, VA, USA), a preferential hyperacuity perimetry task used to detect conversion in patients with AMD [69]—such devices are unlikely to have the same reach as mobile-based applications. These hardware-based devices will be less able to address the short-term need for remote monitoring, accelerated by the COVID-19 pandemic, and longer-term needs related to the advent of treat-and-extend protocols and longer-lasting therapies.

Although this review has identified a growing body of research concerning the application of mobile technologies for monitoring macular health, the current evidence base is limited in its maturity and the extent of supporting evidence varies widely between apps. Some apps have amassed considerable patient numbers, and the SDH test has been investigated in numerous studies spanning the basic science underpinning the development of the test, cross-sectional investigation and real-world implementation. In contrast, other apps have only been tested in one or two studies with relatively few participants and provide supporting evidence on only a select number of indicators of task validity (Table 3). The majority of studies have been cross-sectional. The evidence base for all apps is currently lacking the robust prospective data needed to truly determine the predictive value of remote monitoring apps, and limited long-term follow-up has made it difficult to accurately determine the true role of remote monitoring in managing disease progression and improving patient outcomes. Most apps have only been tested in feasibility and validation studies. Performance data on the sensitivity and specificity of apps are currently very limited and none have been gained from real-world studies in which apps are used as intended in clinical practice; generating such data should be a priority focus for future studies. Physicians need to be confident that patients with disease or without disease are correctly identified so that additional burden is not placed on clinics and patients – if a patient is called into the clinic it must be warranted, and if a patient is not called into the clinic it must be because disease or additional disease progression is truly absent. To instil this confidence, real-world, pragmatic studies are needed, in addition to validation studies. The fact that validation studies are the dominant source of data also means that clinicians cannot yet gain a clear sense of whether these apps are most appropriate for use in detecting new disease onset, and thus initiating prompt treatment, or for ongoing monitoring of disease progression in order to detect reactivation of stable disease and tailor anti-VEGF treatment regimens. Furthermore, the studies identified in this review have examined a varied patient population representing numerous pathologies, including DMO, AMD and other chronic eye conditions. There is currently little directly comparable research across or within apps with which to establish the stability of test performances for any given pathology.

A further benefit of additional real-world studies will be to assess any hardware limitations arising when apps are used on patients’ personal devices. Many of the existing studies of remote monitoring apps provided participants with new mobile devices, or developed apps for use with specific technical specifications (e.g., Apple retina display [48]). Although some apps include features that measure and control ambient light or screen luminance [48, 49], they cannot control for hardware factors, such as screen damage, and not all apps include such features. Results may differ when apps are used on patients’ personal devices, which are likely to vary in age and specification. In addition, patients may encounter device compatibility issues when using older devices, potentially impacting adherence and persistence. Many validation studies used controlled conditions when testing apps, including specifying testing distance and room lighting. Such conditions will be out of researchers’ control in a real-world setting, and even in those apps that have built-in controls (e.g., OdySight) these have not yet been reliably tested.

These early studies have highlighted gaps yet to be addressed when serving the needs of patients with degenerative maculopathies. Most studies have excluded patients with certain neurological and psychological comorbidities such as dementia and Parkinson’s disease: conditions that are likely to occur with some frequency in these elderly patient groups. Such exclusions highlight the fact that remote monitoring will not be a feasible option for all patients and cannot fully replace traditional methods of clinical monitoring. Despite studies reporting positive usability data and favourable responses from patients, researchers have noted the possible difficulties of introducing some patients to digital technologies. It should be noted that studies reporting usability or patient satisfaction data are in the minority, and only one reports rich qualitative data on patient experience [70]. Data from the MONARCH study, obtained from semi-structured interviews, has shown home monitoring methods to be acceptable to patients with nAMD and their caregivers [70]. Obtaining further data of this kind will allow greater understanding of how remote monitoring technologies can be developed and implemented to best meet patient need and maximise engagement. Clinicians should consider that not all patients will be familiar with the use of mobile apps. In particular, the results reported by Chong Teo et al. when implementing remote monitoring to replace appointments deferred during a COVID-19 lockdown emphasise the need to support patients in accessing and engaging with apps [66].

Finally, no cost-effectiveness data were identified for any of the apps included in this review. Once the clinical value of remote monitoring has been established, the next step to ensure completeness of the evidence base will be to investigate cost- effectiveness when used in real-world practice. Unacceptable costs will be a barrier to adoption of technologies that are otherwise effective and acceptable to clinicians and patients.

To address the limitations identified in this review, the field of remote monitoring research must progress and expand. Of the seven apps identified in this review, four have received some form of regulatory clearance. To ensure confidence among clinicians in the use of remote monitoring, it is important that apps are developed to the rigorous standards of clinical testing necessary to confirm clinical validity and meet requirements for obtaining regulatory approval. For clinicians to have confidence in the performance and clinical utility of remote monitoring for patients with macular pathology, studies establishing the sensitivity and specificity of novel tests and further longitudinal studies of remote monitoring as used in real-world clinical practice are required. Careful consideration should be given to supporting patients in downloading and using apps during future research into, and roll-out of, remote monitoring technologies to minimise digital exclusion and maximise the reach of remote monitoring methods.

Although anti-VEGF therapies remain the treatment standard for patients with macular pathology, several novel therapies and delivery mechanisms are in development or have been recently approved that will allow for greater extension of treatment interval and reduction in overall number of clinic visits [71–76]. Technologies such as remote monitoring can be used to further reduce monitoring burden and support the implementation of novel therapies by providing a reliable ‘safety net’ in the extended period between treatment visits. When used together, remote monitoring and longer acting therapies have the potential to enhance the value of one another and provide substantial advances in patient care. Growing the research into remote monitoring methods alongside the development of these novel treatments will hopefully result in robust, evidence-based solutions that reassure patients and physicians between clinic visits and ensure that remote monitoring techniques can be fully integrated into treatment pathways as soon as new therapies are available.

### Summary

#### What was known before

- Numerous apps for remote monitoring of visual function are in development or already available to patients and clinicians.
- Remote vision monitoring has the potential to detect early signs of disease activity or re-activation and thus contribute to prompt initiation of treatment or identify patients requiring earlier in-person clinic visits.
- Wide-spread adoption of home vision monitoring apps requires that they are able to detect subtle changes in visual function and are acceptable and easy to use, particularly for a vulnerable patient population with age-related macular degeneration.
- There is a growing body of literature generating evidence on the sensitivity and specificity of home vision monitoring apps to detect changes in disease activity.
- The level of evidence is variable across different apps and visual function metrics, with shape discrimination hyperacuity-based apps having been more extensively assessed.

#### What this study adds

- The level of evidence is limited in its maturity and the extent of supporting evidence varies widely between apps.
- Most evidence originates from feasibility or validation studies with limited follow-up. More robust evidence exists for a minority of apps spanning basic research, feasibility, validation and real-world studies with longitudinal follow-up.
- Pragmatic real-world studies with longer follow-up periods assessing the performance of such apps in their intended clinical environment should be prioritized in order to provide confidence to patients and clinicians on the utility of these technologies.
- Only two real-world studies have so far been conducted demonstrating the feasibility of deployment at scale, as well as the challenges around usability and the need to mitigate against risks of digital exclusion.
- The reported positive usability results is caveated with the need to expand inclusivity for all patient populations, with a significant proportion of patients encountering challenges in independent use of the apps.
- One study presented rich qualitative data on factors influencing the usability of home vision monitoring apps; further such work will be crucial for devising strategies to maximize digital inclusion.
- Seven apps were identified in this review, of which four have some level of regulatory approval.
- For emerging technologies, validation studies are warranted to fulfill regulatory requirements and real-world implementation studies are essential to generate confidence in patients and clinicians and address identified barriers to usability.
- The advent of longer-lasting treatments for common retinal disease alongside use of robustly validated apps and thoroughly assessed for usability has the potential of providing a “safety net” for patients while reducing the frequency of hospital appointments.

## References

[1] Schmidt-Erfurth U, Chong V, Loewenstein A, Larsen M, Souied E, Schlingemann R (2014). Guidelines for the management of neovascular age-related macular degeneration by the European Society of Retina Specialists (EURETINA). Br J Ophthalmol.

[2] Wykoff CC, Clark WL, Nielsen JS, Brill JV, Greene LS, Heggen CL (2018). Optimizing anti-VEGF treatment outcomes for patients with neovascular age-related macular degeneration. J Manag Care Spec Pharm.

[3] Schmidt-Erfurth U, Garcia-Arumi J, Bandello F, Berg K, Chakravarthy U, Gerendas BS (2017). Guidelines for the management of diabetic macular edema by the European Society of Retina Specialists (EURETINA). Ophthalmologica.

[4] Do DV, Gower EW, Cassard SD, Boyer D, Bressler NM, Bressler SB (2012). Detection of new-onset choroidal neovascularization using optical coherence tomography: the AMD DOC study. Ophthalmology.

[5] Parikh R, Avery RL, Saroj N, Thompson D, Freund KB (2019). Incidence of new choroidal neovascularization in fellow eyes of patients with age-related macular degeneration treated with intravitreal aflibercept or ranibizumab. JAMA Ophthalmol.

[6] Ciulla TA, Amador AG, Zinman B (2003). Diabetic retinopathy and diabetic macular edema: pathophysiology, screening, and novel therapies. Diabetes Care.

[7] Adamis AP, Brittain CJ, Dandekar A, Hopkins JJ (2020). Building on the success of anti-vascular endothelial growth factor therapy: a vision for the next decade. Eye.

[8] Wubben TJ, Johnson MW (2019). Anti-vascular endothelial growth factor therapy for diabetic retinopathy: Consequences of inadvertent treatment interruptions. Am J Ophthalmol.

[9] Kim JH, Chang YS, Kim JW (2017). Natural course of patients discontinuing treatment for age-related macular degeneration and factors associated with visual prognosis. Retina.

[10] Khanna S, Komati R, Eichenbaum DA, Hariprasad I, Ciulla TA, Hariprasad SM (2019). Current and upcoming anti-VEGF therapies and dosing strategies for the treatment of neovascular AMD: a comparative review. BMJ Open Ophthalmol.

[11] Malasinghe LP, Ramzan N, Dahal K (2019). Remote patient monitoring: a comprehensive study. J Ambient Intell Humaniz Comput.

[12] Vegesna A, Tran M, Angelaccio M, Arcona S (2017). Remote patient monitoring via non-invasive digital technologies: a systematic review. Telemed J E Health.

[13] Pew Research Center. Mobile fact sheet. 2021. https://www.pewresearch.org/internet/fact-sheet/mobile/ (Accessed 02 February 2022).

[14] Statista.com. Share of adults who own a smartphone in the United Kingdom (UK) in 2008 and 2019 to 2020, by demographics. 2020. https://www.statista.com/statistics/956297/ownership-of-smartphones-uk/ (Accessed 02 February 2022).

[15] Martiniello N, Eisenbarth W, Lehane C, Johnson A, Wittich W (2019). Exploring the use of smartphones and tablets among people with visual impairments: Are mainstream devices replacing the use of traditional visual aids?. Assist Technol.

[16] Ludwig C, Callaway N, Ho Park J, Leng T (2016). Mobile health in the retinal clinic population: access to and interest in self-tracking. Ophthalmic Surg Lasers Imaging Retin.

[17] Chopra R, Wagner SK, Keane PA (2021). Optical coherence tomography in the 2020s—outside the eye clinic. Eye.

[18] Liu Y, Holekamp NLM, Heier JS. Prospective, longitudinal study: daily self- imaging with home OCT in neovascular age-related macular degeneration. Ophthalmol Retina. 2022;S2468-6530 (2422)00073-00072. 10.1016/j.oret.2022.02.011. 10.1016/j.oret.2022.02.011 35240337

[19] Charlesworth JM, Davidson MA (2019). Undermining a common language: smartphone applications for eye emergencies. Med Devices (Auckl).

[20] Steren BJ, Young B, Chow J (2021). Visual acuity testing for telehealth using mobile applications. JAMA Ophthalmol.

[21] Yeung WK, Dawes P, Pye A, Charalambous A-P, Neil M, Aslam T (2019). eHealth tools for the self-testing of visual acuity: a scoping review. NPJ Digit Med.

[22] Chen JS, Adelman RA (2016). Hyperacuity exam screens for choroidal neovascularization in age-related macular degeneration on a mobile device. Ophthalmic Surg Lasers Imaging Retin.

[23] Schmid MK, Thiel MA, Lienhard K, Schlingemann RO, Faes L, Bachmann LM (2019). Reliability and diagnostic performance of a novel mobile app for hyperacuity self-monitoring in patients with age-related macular degeneration. Eye.

[24] Wang Y-Z, He Y-G, Mitzel G, Zhang S, Bartlett M (2013). Handheld shape discrimination hyperacuity test on a mobile device for remote monitoring of visual function in maculopathy. Invest Ophthalmol Vis Sci.

[25] Winther C, Frisén L (2015). New rarebit vision test captures macular deficits hidden to acuity tests. Acta Ophthalmol.

[26] Lovie-Kitchin J, Feigl B (2005). Assessment of age-related maculopathy using subjective vision tests. Clin Exp Optom.

[27] Ismail GM, Whitaker D (1998). Early detection of changes in visual function in diabetes mellitus. Ophthalmic Physiol Opt.

[28] Stavrou EP, Wood JM (2003). Letter contrast sensitivity changes in early diabetic retinopathy. Clin Exp Optom.

[29] Schwartz R, Loewenstein A (2015). Early detection of age related macular degeneration: current status. Int J Retin Vitreous.

[30] Crossland M, Rubin G (2007). The Amsler chart: absence of evidence is not evidence of absence. Br J Ophthalmol.

[31] Liu L, Wang Y-Z, Bedell HE (2014). Visual-function tests for self-monitoring of age- related macular degeneration. Optom Vis Sci.

[32] Faes L, Bodmer NS, Bachmann LM, Thiel MA, Schmid MK (2014). Diagnostic accuracy of the Amsler grid and the preferential hyperacuity perimetry in the screening of patients with age-related macular degeneration: systematic review and meta-analysis. Eye.

[33] Schuchard RA (1993). Validity and interpretation of Amsler grid reports. Arch Ophthalmol.

[34] Fine AM, Elman MJ, Ebert JE, Prestia PA, Starr JS, Fine SL (1986). Earliest symptoms caused by neovascular membranes in the macula. Arch Ophthalmol.

[35] Keane PA, de Salvo G, Sim DA, Goverdhan S, Agrawal R, Tufail A (2015). Strategies for improving early detection and diagnosis of neovascular age-related macular degeneration. Clin Ophthalmol.

[36] Zaidi FH, Cheong-Leen R, Gair EJ, Weir R, Sharkawi E, Lee N (2004). The Amsler chart is of doubtful value in retinal screening for early laser therapy of subretinal membranes. West Lond Surv Eye.

[37] Midena E, Degli Angeli C, Blarzino MC, Valenti M, Segato T (1997). Macular function impairment in eyes with early age-related macular degeneration. Invest Ophthalmol Vis Sci.

[38] Phipps JA, Guymer RH, Vingrys AJ (2003). Loss of cone function in age-related maculopathy. Invest Ophthalmol Vis Sci.

[39] Stangos N, Voutas S, Topouzis F, Karampatakis V (1995). Contrast sensitivity evaluation in eyes predisposed to age-related macular degeneration and presenting normal visual acuity. Ophthalmologica.

[40] Westheimer G, McKee SP (1977). Integration regions for visual hyperacuity. Vis Res.

[41] Westheimer G, McKee SP (1977). Spatial configurations for visual hyperacuity. Vis Res.

[42] Wilkinson F, Wilson HR, Habak C (1998). Detection and recognition of radial frequency patterns. Vis Res.

[43] Wang YZ, Wilson E, Locke KG, Edwards AO (2002). Shape discrimination in age- related macular degeneration. Invest Ophthalmol Vis Sci.

[44] Pitrelli Vazquez N, Knox PC (2015). Assessment of visual distortions in age-related macular degeneration: emergence of new approaches. Br Ir Orthopt J.

[45] Wang Y-Z (2001). Effects of aging on shape discrimination. Optom Vis Sci.

[46] Frisén L (2002). New, sensitive window on abnormal spatial vision: rarebit probing. Vis Res.

[47] Nilsson M, von Wendt G, Wanger P, Martin L (2007). Early detection of macular changes in patients with diabetes using Rarebit Fovea Test and optical coherence tomography. Br J Ophthalmol.

[48] Winther C, Frisén L. Self-testing of vision in age-related macula degeneration: A longitudinal pilot study using a smartphone-based rarebit test. J Ophthalmol. 2015:285463. 10.1155/2015/285463. 10.1155/2015/285463 PMC4466471 26124958

[49] Brucker J, Bhatia V, Sahel J-A, Girmens J-F, Mohand-Saïd S (2019). Odysight: a mobile medical application designed for remote monitoring—a prospective study comparison with standard clinical eye tests. Ophthalmol Ther.

[50] Nikam A, Lefumat H, Grondin E, Bhatia V (2020). OdySight: real-world data analysis of a mobile medical application for ophthalmology. J Ophthalmol Sci.

[51] Khurana RN, Hoang C, Khanani AM, Steklov N, Singerman LJ (2021). A smart mobile application to monitor visual function in diabetic retinopathy and age-related macular degeneration: The CLEAR study. Am J Ophthalmol.

[52] Wu Z, Guymer RH, Jung CJ, Goh JK, Ayton LN, Luu CD, et al. Measurement of retinal sensitivity on tablet devices in age-related macular degeneration. Transl Vis Sci Technol. 2015;4. 10.1167/tvst.1164.1163.1113. 10.1167/tvst.4.3.13 PMC4497484 26175959

[53] Ho CYD, Wu Z, Turpin A, Lawson DJ, Luu C, McKendrick A, et al. A tablet- based retinal function test in neovascular age-related macular degeneration eyes and at-risk fellow eye. Transl Vis Sci Technol. 2018;7. 10.1167/tvst.1167.1162.1162. 10.1167/tvst.7.2.2 PMC5837668 29520334

[54] Food and Drug Administration. 510(k) summary: K121738 2012. https://www.accessdata.fda.gov/cdrh_docs/pdf12/K121738.pdf (Accessed 02 February 2022).

[55] Ku JY, Milling AF, Pitrelli Vazquez N, Knox PC (2016). Performance, usability and comparison of two versions of a new macular vision test: the handheld Radial Shape Discrimination test. PeerJ.

[56] Kaiser PK, Wang YZ, He YG, Weisberger A, Wolf S, Smith CH (2013). Feasibility of a novel remote daily monitoring system for age-related macular degeneration using mobile handheld devices: results of a pilot study. Retina.

[57] Korot E, Pontikos N, Drawnel FM, Jaber A, Fu DJ, Zhang G (2022). Enablers and barriers to deployment of smartphone-based home vision monitoring in clinical practice settings. JAMA Ophthalmol.

[58] Lott LA, Schneck ME, Haegerstrom-Portnoy G, Hewlett S, Stepien-Bernabe N, Gauer BM (2021). Simple vision function tests that distinguish eyes with early to intermediate age-related macular degeneration. Ophthalmic Epidemiol.

[59] Pitrelli Vazquez N, Harding SP, Heimann H, Czanner G, Knox PC (2018). Radial shape discrimination testing for new-onset neovascular age-related macular degeneration in at-risk eyes. PLoS One.

[60] Schneck ME, Lott LA, Haegerstrom-Portnoy G, Hewlett S, Gauer BM, Zaidi A (2021). Visual function in eyes with intermediate AMD with and without retinal pigment abnormalities. Optom Vis Sci.

[61] Wang Y-Z, He Y-G, Csaky KG, Mitzel G, Hernandez K, Zhang S (2015). Diabetic retinopathy and the myVisionTrack® app (DRAMA) study. Invest Ophthalmol Vis Sci.

[62] Wang Y-Z, He Y-G, Mitzel G, Zhang S, Bartlett MB (2014). Compliance and test variability of patients with maculopathy in using an iphone-based shape discrimination hyperacuity test at home. Invest Ophthalmol Vis Sci.

[63] Wang Y-Z, Mitzel G (2013). A new contour integration macular perimetry (CIMP) on iPad for visual function evaluation in maculopathy. Invest Ophthalmol Vis Sci.

[64] Wang Y-Z, Ying G-S, Mitzel G, He Y-G (2011). Sensitivity and specificity of shape discrimination hyperacuity for differentiating exudative AMD from moderate AMD. Invest Ophthalmol Vis Sci.

[65] Ward E, Wickens RA, O’Connell A, Culliford LA, Rogers CA, Gidman EA (2021). Monitoring for neovascular age-related macular degeneration (AMD) reactivation at home: the MONARCH study. Eye.

[66] Chong Teo KY, Bachmann LM, Sim D, Yen LS, Tan A, Wong TY (2021). Patterns and characteristics of a real-world implementation of a self-monitoring program for retina diseases during COVID-19 pandemic. Ophthalmol. Retina.

[67] Gangwani RA, Leung CK, Lam PT (2012). Full correction of refractive errors during automated perimetry: does it really matter? Hong Kong. J Ophthalmol.

[68] Zhao C, Cun Q, Tao Y-J, Yang W-Y, Zhong H, Li F-J (2020). Effect of intraocular lens implantation on visual field in glaucoma and comorbid cataracts. Int J Ophthalmol.

[69] Chew EY, Clemons TE, Bressler SB, Elman MJ, Danis RP, Domalpally A (2014). Randomized trial of the ForeseeHome monitoring device for early detection of neovascular age-related macular degeneration. The HOme Monitoring of the Eye (HOME) study design — HOME Study report number 1. Contemp Clin Trials.

[70] O’Connor SR, Treanor C, Ward E, Wickens RA, O’Connell A, Culliford LA (2022). Patient acceptability of home monitoring for neovascular age-related macular degeneration reactivation: a qualitative study. Int J Environ Res Public Health.

[71] Campochiaro PA, Marcus DM, Awh CC, Regillo C, Adamis AP, Bantseev V (2019). The port delivery system with ranibizumab for neovascular age-related macular degeneration: Results from the randomized phase 2 ladder clinical trial. Ophthalmology.

[72] Ellis S, Buchberger A, Holder J, Orhan E, Hughes J (2020). GT005, a gene therapy for the treatment of dry age-related macular degeneration (AMD). Invest Ophthalmol Vis Sci.

[73] Khanani AM, Patel SS, Ferrone PJ, Osborne A, Sahni J, Grzeschik S (2020). Efficacy of every four monthly and quarterly dosing of faricimab vs ranibizumab in neovascular age-related macular degeneration: The STAIRWAY phase 2 randomized clinical trial. JAMA Ophthalmol.

[74] Khanani AM, Callanan D, Dreyer R, Chen S, Howard JG, Hopkins JJ (2021). End-of-study results for the Ladder phase 2 trial of the port delivery system with ranibizumab for neovascular age-related macular degeneration. Ophthalmol Retin.

[75] Dugel PU, Koh A, Ogura Y, Jaffe GJ, Schmidt-Erfurth U, Brown DM (2020). HAWK and HARRIER: Phase 3, multicenter, randomized, double-masked trials of brolucizumab for neovascular age-related macular degeneration. Ophthalmology.

[76] Khurana RN, Rahimy E, Joseph WA, Saroj N, Gibson A, Vitti R (2019). Extended (every 12 weeks or longer) dosing interval with intravitreal aflibercept and ranibizumab in neovascular age-related macular degeneration: post hoc analysis of view trials. Am J Ophthalmol.

